# A New Method of Secure Authentication Based on Electromagnetic Signatures of Chipless RFID Tags and Machine Learning Approaches

**DOI:** 10.3390/s20216385

**Published:** 2020-11-09

**Authors:** Dragoș Nastasiu, Răzvan Scripcaru, Angela Digulescu, Cornel Ioana, Raymundo De Amorim, Nicolas Barbot, Romain Siragusa, Etienne Perret, Florin Popescu

**Affiliations:** 1 Military Technical Academy, Department of Communications and Military Electronic Systems, 050141 Bucharest, Romania; razvan.scripcaru@mta.ro (R.S.); angela.digulescu@mta.ro (A.D.); florin.popescu@mta.ro (F.P.); 2 Gipsa-lab, Université Grenoble Alpes, 38402 Grenoble, France; cornel.ioana@gipsa-lab.grenoble-inp.fr; 3 Grenoble INP, LCIS, Université Grenoble Alpes, 26902 Valence, France; raymundo.de-amorim-junior@lcis.grenoble-inp.fr (R.D.A.J.); nicolas.barbot@lcis.grenoble-inp.fr (N.B.); romain.siragusa@lcis.grenoble-inp.fr (R.S.); etienne.perret@lcis.grenoble-inp.fr (E.P.)

**Keywords:** chipless RFID tags, classification, authentication, machine learning, electromagnetic signature, data augmentation, python, keras

## Abstract

In this study, we present the implementation of a neural network model capable of classifying radio frequency identification (RFID) tags based on their electromagnetic (EM) signature for authentication applications. One important application of the chipless RFID addresses the counterfeiting threat for manufacturers. The goal is to design and implement chipless RFID tags that possess a unique and unclonable fingerprint to authenticate objects. As EM characteristics are employed, these fingerprints cannot be easily spoofed. A set of 18 tags operating in V band (65–72 GHz) was designed and measured. V band is more sensitive to dimensional variations compared to other applications at lower frequencies, thus it is suitable to highlight the differences between the EM signatures. Machine learning (ML) approaches are used to characterize and classify the 18 EM responses in order to validate the authentication method. The proposed supervised method reached a maximum recognition rate of 100%, surpassing in terms of accuracy most of RFID fingerprinting related work. To determine the best network configuration, we used a random search algorithm. Further tuning was conducted by comparing the results of different learning algorithms in terms of accuracy and loss.

## 1. Introduction

Nowadays, radio frequency (RF) identification (RFID) is used in various applications such as: object identification [1], tracking [2], and access control [3]. However, in the literature, it has been described that conventional RFID has considerable potential in solving authentication threats such as counterfeiting [4]. Authentication in the global supply chains offers many opportunities to assess the products authenticity. Three security levels are usually considered [5,6]: overt, that relates visible characteristics, covert or hidden markers (medium security level), and forensic techniques (high security level). Some of the overt authentication techniques used in supply chains include holograms, color-shifting inks, security threads, watermarks, and sequential product numbering. Covert technologies include similar elements such as security inks and invisible printing. The main disadvantage of these methods is that the unique identifier can be cloned. They are difficult to apply to specific categories of goods if they are too small (i.e., an electronic circuit) or if they are used in extreme environmental conditions (i.e., aircraft components). A more secure approach implies using forensic features and RFID technology. In order to provide a high security level authentication process, a database of the electromagnetic (EM) response of each tag is needed. The evaluation of the richness of the EM fingerprint information is an essential point for the application. The database enrolment is carried out after the manufacturing process. The neural network we propose authenticates an unknown tag, based on the characteristics it learnt in the training phase, thus aiming to provide a secure authentication method to combat the counterfeit in the supply chain environment.

The methods based on the identification of individual transmitters/receivers or objects by using the EM signal characterization are called RF fingerprint identifications. These methods attempt to extract an identifier from the received signal. The RF fingerprint identification exploits the intrinsic characteristics related to hardware and manufacturing randomness, which can be extracted by processing the transient round-trip signal [7].

Traditional passive UHF RFID systems can be adapted to authentication solutions. Some protocols are proposed to improve its security level [6]. Physical attributes of back-scattered signals from the RFID tags can be used for authentication applications [7,8,9,10,11,12,13,14,15]. However, this functionality leads to additional blocks in the ASIC implementation, which increases the power consumption, reduces the reading distance, and significantly increases the final cost of the application.

**Figure 1:**
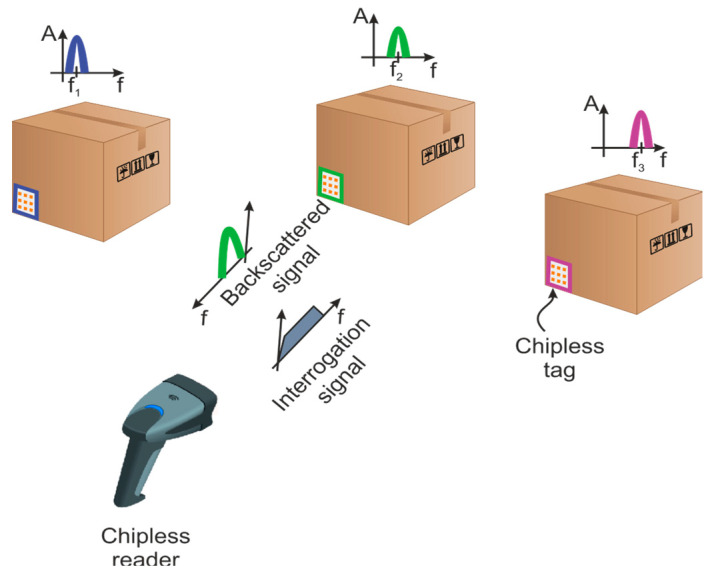
Basic chipless radio frequency identification (RFID) system. Each tag has a different electromagnetic (EM) characteristic related to manufacture randomness.

Chipless RFID technology is an intermediate technology between the barcodes and the UHF RFID [16]. It combines some features of the barcodes and the UHF RFID. Instead of storing the identifier in an IC, as in the case of UHF RFID, the information (i.e., the EM response) is directly linked to the geometry of the printed elements. In this sense, the chipless tags can be seen as a radar target designed to scatter a specific electromagnetic signature. In general, chipless tags cannot change their information once manufactured. In identification applications, as in our case, the backscattered signal can be exploited in different ways, for instance by considering the amplitude [17], the frequency [18], the phase [19], or some combination of these parameters. Two main families of tags can be distinguished according to the technique used to code information: the time domain tag [20,21,22] and the frequency domain tag [23]. Time coding is based on a reflectometry principle. The reader listens to the backward echoes from the sent pulse. For the frequency coding, the information is coded in the resonant frequency of each resonator. For that, a pulse that covers the entire spectrum is sent by the reader to excite every resonator of the tag. The identifier is the round-trip signal that is received by the reader. A basic chipless RFID system is depicted in Figure 1. The resonant patterns show dips/peaks in the spectrum of the backscatter signal. Considering the frequency approach, binary information can be associated with the presence/absence or shifts of a given frequency.

**Figure 2:**
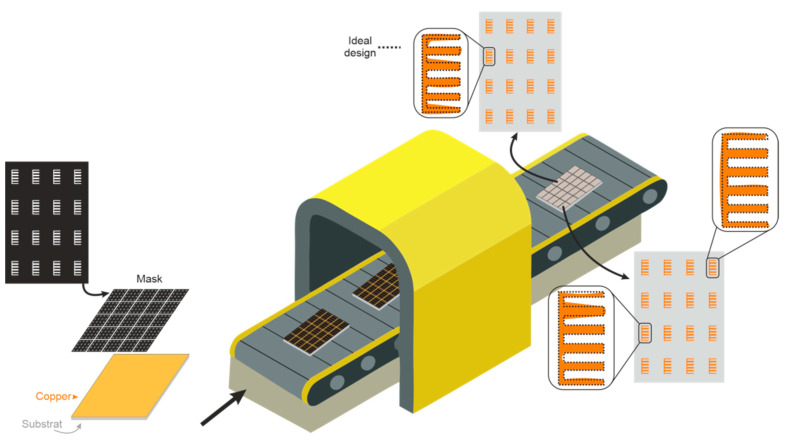
Dimensional inhomogeneities caused by a manufacturing process.

The authentication using chipless RFID tags in ultra-wide band (UWB) (3.1–10.6 GHz) was initially demonstrated in [24]. The concept of chipless authentication is based on the idea that it is extremely difficult to identically reproduce materials (tags) that naturally have random characteristics due to the manufacturing process [25,26,27]. The natural dimensional variations in the chipless tags realizations lead to singular variations on the RF backscatter response, which paves the way to the high-level security applications. Moreover, the cloning of chipless tags in the proposed method is very difficult; since a nonintrusive approach is employed and the geometrical characteristics of the tags cannot be copied by examining the surface, neither can the RF signal be easily spoofed. The proposed concept of authentication using the natural randomness is much vaster than the chemical etching process example (over/under etching). As depicted in Figure 2, it includes the natural variations of the substrate permittivity, the copper thickness, or the mask film. For instance, the tag mask is designed and, after the treatment, the supposed ideal tags pattern is etched on the substrate. As noted in the inset of Figure 2, geometric inherent elements variations are introduced by the manufacturing process, which produce randomness over the geometric patterns of the elements. We propose to use the V-band especially because of its smaller wavelengths that are more sensitive to geometrical randomness, thus generating unique EM identifiers for each fabricated tag. The frequency band and the geometry of tags do not affect the training process of the proposed neural network as long as the EM signatures are differentiable. The inherent randomness in the fabrication process ensures that the tags are unique. To highlight the uniqueness, we use higher frequencies (V band) to characterize the back-scattered response of the tag. Therefore, the limitations regarding the number of tags are related to the frequency resolution of the EM signature. If we have a good resolution, the neural network can be fitted properly, otherwise the results will not be as desired.

In [28], various methods of classifications were examined in order to effectively differentiate between the identities (i.e., the EM signature) of chipless RFID tags. Firstly, as a supervised method, linear discriminant analysis (LDA) [29] was chosen based on its ability to focus on the most discriminating features between classes. The study in [28] demonstrated that this technique can reach a high recognition rate regardless of the sampling resolution of the EM signature (400/12,800 samples) and the number of tag classes (5/10/20 classes). Furthermore, the performances of unsupervised classifications were evaluated using multiple distance measures such as: Euclidean, Manhattan, Lorentzian, and the normalized correlation coefficient. Classifications using other techniques were also studied: dynamic time warping (DTW) [30] and computing the Manhattan distance between two tag measurements after performing wavelet transformation (WT) and comparing the resultant coefficients [31]. However, the methods presented until now have some disadvantages, namely regarding the lower recognition rate, which is presented later on, in Section 3. The simplicity of a neural network opens new doors to having the fully trained model transformations in an IC, which can be used in RFID fingerprinting equipment. The drawback of the aforementioned techniques that pushed us to analyze the ML approach is their inability to distinguish between similar data. ML successfully breaks this barrier and offers a lot of opportunities to exploit.

Neural networks come in different shapes and forms mostly derived from architectures like: Multilayer Perceptron (MLP), Convolutional Neural Network (CNN) and Recurrent Neural Network (RNN). These networks are tweaked and modified in order to solve a particular problem. As a consequence, there are a large variety of models to choose from and to analyze, with each one of them being suited for a narrow area of applications, such as pattern recognition, trend detection, and optimization problems [32]. These networks have the capability to characterize complex or inaccurate data, thus exceeding the decision capabilities of other processing techniques [33]. The main design purpose is to find unique identifiers for each V-band operating tag and to validate the ML approach in an authentication task, to combat the extent of the counterfeiting phenomena. For clarity, we summarize our research and contributions as follows:

- We propose chipless RFID tags operating in V-band, which are more sensitive to geometrical inhomogeneities than other bands at lower frequencies. In order to harness the fabrication randomness, EM signatures are employed to characterize each tag.
- We evaluate the capacity of a neural model to identify and authenticate the EM signatures of the tags in a supply chain scenario and we obtained a high recognition rate.

The paper is organized as follows: Section 2 provides the proof of concept for V-band applications and describes the chipless RFID technology used in this work. In the second part of the section, a description of the dataset acquisition environment and details regarding the architecture and the machine learning (ML) approach used to validate the authentication method are given. Section 3 presents the analysis of the experimental results and a performance comparison of the techniques found in the literature. Section 4 draws the conclusions of this paper.

## 2. Materials and Methods

### 2.1. Proof of Concept to V-Band Applications

The chipless tags can be seen as a radar target designed to scatter a specific electromagnetic signature. In chipless RFID applications, it is possible to authenticate such items with RFID chipless tags using a noninvasive method based on the exploitation of a distinctive signature, which paves the way to authenticate documents (printing the tag directly on paper) or even specific products. The tags’ structure is basically composed of metallic ink printed on a substrate; therefore, no components are needed. Nowadays, a chipless application designed to operate in the X-band (8–12 GHz) was reported [34].

**Figure 3:**
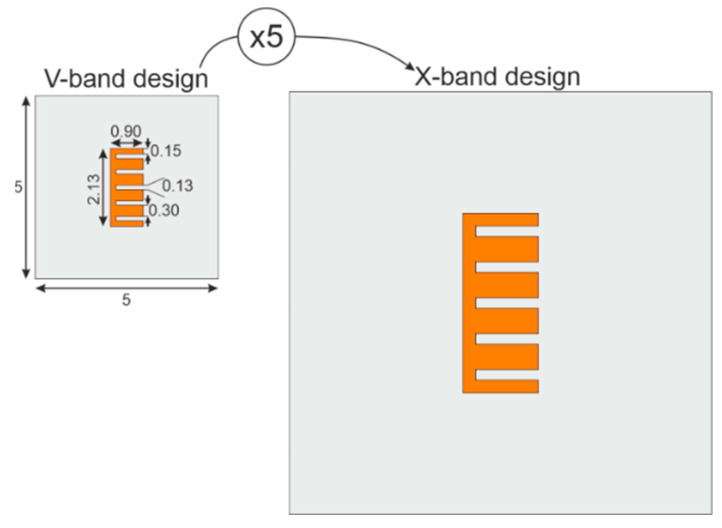
E-shaped chipless resonator designed to different bands (V-band and X-band), all dimensions are in millimeter.

In the interest of increasing the sensibility due to manufacturing intrinsic errors, a chipless millimeter-wave E-shape resonator was designed and simulated for proof of concept. The fabricated tag is further used to validate the better distinctiveness in V-band. Contrary to X-band RF approaches, the V-band range (50–75 GHz) is chosen to grow the tag’s sensitivity related to uncertainties in the fabrication process. Then, for comparison purposes, the E-shaped V-band geometrical dimensions are scaled 5 times generating the X-band design, as shown in Figure 3.

**Figure 4:**
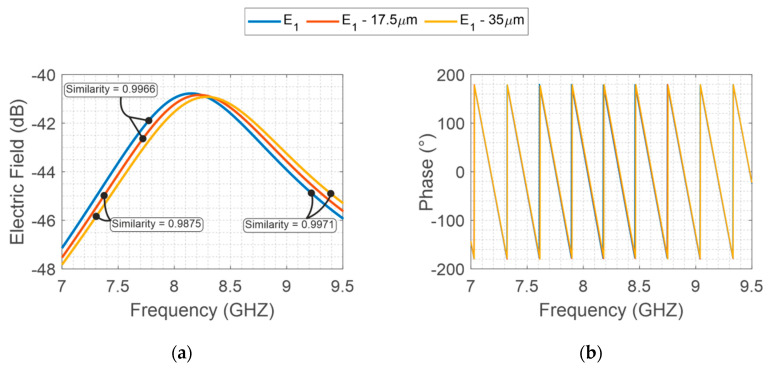
E-Shaped X-band backscattered EM field, (**a**) S_21_ magnitude and (**b**) phase considering the geometrical variations (17.5 μm and 35 μm).

**Figure 5:**
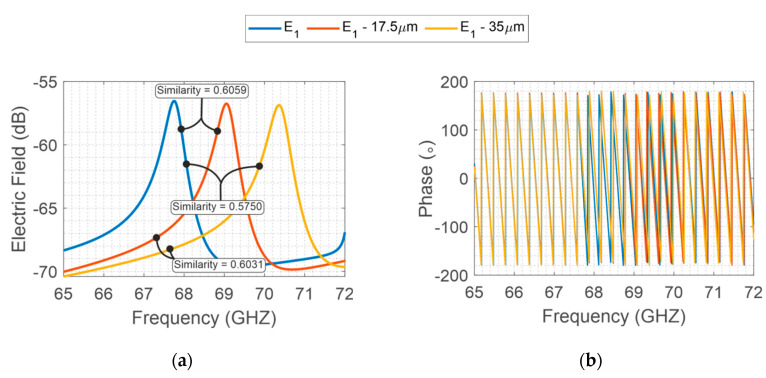
E-Shaped V-band backscattered EM field, (**a**) S_21_ magnitude and (**b**) phase, whereas the E_V_ represents the backscattered signal from the V-band E-shape resonator.

Considering the same manufacturing method, the same precision is imposed for both structures. For instance, for photolithograph methods, a precision around 35 μm is provided. In this method, the main geometrical errors in the designed structures are due to over-etching caused during the fabrication process. Then, to highlight the geometrical variation on the backscattered resonator response, specific variations are imposed on the two designs. An interrogation plane wave is sent in far-field configuration towards the tag, and then the backscatter S_21_ is recovered by a far-field probe. As depicted in Figure 4, the intentional variations of 17.5 μm and 35 μm on X-band structure provide a minor electromagnetic signal variation when compared to V-band (Figure 5). Furthermore, for better comprehension, the similarities among the signals are evaluated, whereas values near 1 represent a high similarity between the compared signals and, values equal to 0 represent different signals.

Therefore, as the randomness is the same for the fabrications and considering the operating range of each structure, the fabrication process imposes a manufacturing error on the structures. Consequently, the structure developed to operate on V-band is more sensitive to the physical error process, resulting in better differentiation between the analyzed signals on the V-band.

Finally, to resume the better distinctiveness due to V-band operation, Table 1 shows all the similarity values associated with each signal pair comparison. Thus, the V-band presents significantly lower similarity coefficients compared to X-band coefficients. In the next sections, a specific V-band chipless tag is presented.

### 2.2. Chipless RFID Tags

**Figure 6:**
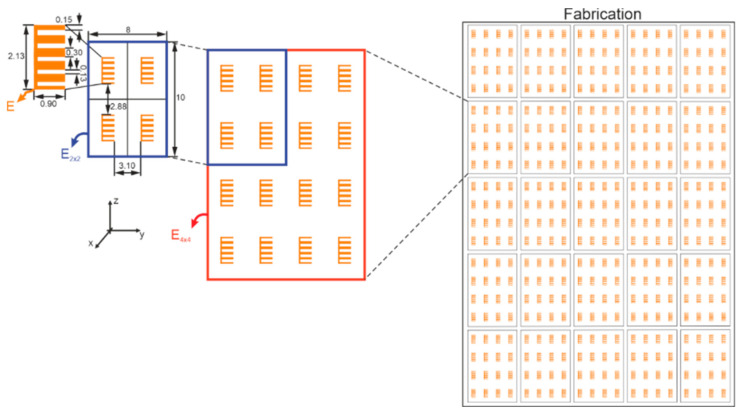
Fabricated chipless tags sharing the same substrate (all dimensions are in millimeters).

A set of 18 tags was fabricated on the same substrate, as shown in Figure 6. It is important to note that all tags come from the same digital file, the same substrate, and these tags share the same mask and fabrication process. In V-band, compared to the X-band, the main limitations are the decrease of the resonator Q factor and the presence of a low radar cross section (RCS) level. The RCS of this scatterer can be difficult to measure in a real environment. Therefore, to overcome this problem, the same scatterer is reproduced several times identically. Thus, to increase the RCS level at a given frequency, the basic principle is to include the same resonator several times on the same tag surface, as depicted in Figure 6.

**Figure 7:**
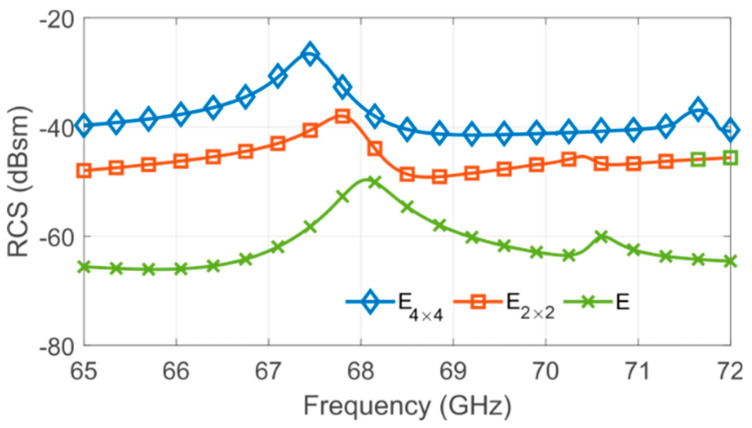
Simulated radar cross section (RCS) versus frequency of different group resonators, where $E_{a\times b}$$E_{a\times b}$ concern the lines *a* and columns *b* of the tag, respectively.

Furthermore, this increase in the number of resonators will carry out a coupling increment between the resonators that may grow the degree of randomness. Due to coupling, the multiplication of the number of scatterers does not carry a proportional augmentation of the RCS level; hence an optimization step is performed. The tag’s EM responses were obtained by EM simulation using CST Microwave Studio. The substrate is Rogers RT5880 with tanδ=0.005, permittivity $\varepsilon_{r}=2.33$, and a thickness of 0.127 mm. Firstly, five C-shape scatterers are put together without spacing between them, forming the E-structure (E in Figure 6). The quality factor *Q* of this E-structure is 84. It is high when compared with [24,34]. It is an important metric in this context since a higher *Q* represents greater sensitivity due to structural variations. However, whatever its quality factor is, tags must have an RCS greater than −40 dB to be read correctly. Then, the E shape scatterer is a better candidate for authentication purposes, and a 4×4 arrangement is adopted to increase the RCS level. The final dimensions of the $E_{4\times 4}$ can be seen in Figure 6. Therefore, the multiplication of the number of resonators leads to a high coupling among the E-structures, which provides a frequency shift along the backscattered responses, as is shown in Figure 7. It is noteworthy that an increase of RCS level does not carry a lower quality factor.

**Figure 8:**
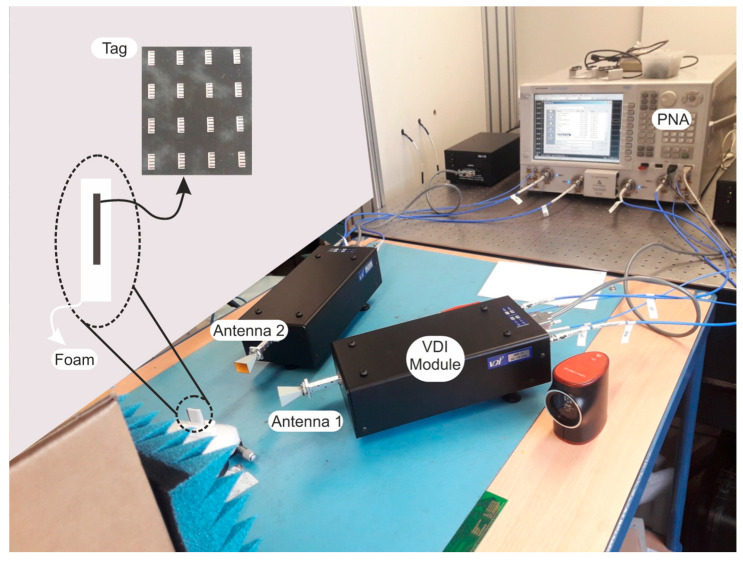
Setup for V-band measurements in office environment.

The dataset was generated in a laboratory as shown in Figure 8. The setup used for data measurement was designed to resemble a scenario in supply chain authentication mechanisms, where the tag has direct visibility with the reader and the authentication method is engaged to determine its authenticity. The direct visibility refers to the fact that the tag is not integrated into a product or packaged and it is measured in an ideal manner. For practical applications, the positions of the tag, the object and the antennas must be stored. The test-bench can be disassembled; however, for measurement reproducibility, the previous positions must be applied. In this way, the object to authenticate is judiciously placed inside the authentication device, whereas the measurement is collected for comparison purposes. The usage of the V band limits the distance of operability and thus, a scenario where tags and readers are in different locations is not very reliable. Moreover, a greater distance increases the possibility of having interferences and artefacts added to the back-scattered signal that make the authentication harder to achieve.

The measurements were performed with an Agilent N5222A (0.01 GHz–26.5 GHz, Keysight Technologies, Santa Rosa, CA, USA) PNA with Virginia Extensions (VDI modules) to operate from 65 GHz to 72 GHz. The VDI module is a frequency multiplier combined with a mixer with a WR_15_ waveguide output connected to horn antennas in copolarization configuration. The tag is positioned inside a thin piece of foam. The dedicated foam substrate, as shown in Figure 8, has been fabricated to significantly reduce the positioning error. The tags were placed at a minimum distance of 15 cm from the antennas and a bi-static configuration with time-gating is used to reduce the multipath interferences and to decrease the clutter contributions. Each tag was measured 10 times in the manner described, thus creating our database. The used set of tags claims the proof of concept for the millimeter-wave chipless tag authentication applications.

One problem that arises is related to how the training of the neural system should be conducted in a real environment. Fitting the neural model should be done prior to deployment (after the manufacturing process) and it should use measurements that correspond to a real scenario where the tags are incorporated into products, like pharmaceuticals products, consumer goods, apparel, spare parts, luxury products, etc. This is essential in order to minimize the probability of misclassification, because integrating tags into products implies modeling the environment noise and interferences into the EM signature. The ideally measured tags are only used to validate the proposed concept. The maximum differentiation among the tags is affected mainly due to two factors: the uncertainty of the manufacturing process, that directly relates to the tag’s back-scattered signal, and the frequency differentiation that concerns the frequency operation of the chipless tag. Therefore, the millimeter-wave operation improves the frequency resolution of the approach (i.e., small geometrical variations result in a significant frequency shift when compared to low-frequency operating tags). For instance, considering the UWB frequency band, the maximum frequency shift seen in the measurements is 80 MHz [34]. On the other hand, for millimeter-wave chipless tags we have around 2 GHz. It is noteworthy, up to now, that is the first time that millimeter-wave resonant chipless tag is reported for authentication purposes.

### 2.3. Neural Network

**Figure 9:**
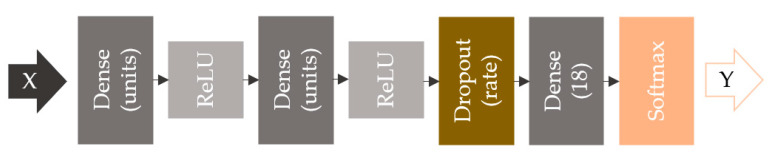
Neural network with two dense layers with ReLU activations, one dropout layer, and SoftMax loss function.

The evaluated network is a 2-layer MLP with ReLU activations and one final dropout layer, as shown in Figure 9. The motivation behind this stands not only in the ease of implementation but also in the advantages this model has: it is flexible, and it can be generally used to classify nonlinear data. The input layer feeds the network EM signatures, $X\in {\mathbb{R}}^{10000}$. The fully connected layers use ReLU activation, which has gain scope because of its simplicity, its accelerated rate of convergence when computing gradients, and its characteristic of not saturating the positive gradients [35]. The regularization layer applies dropout with a rate of 0.3 (fraction of the input units to drop), which leads to reduced overfitting by making the presence of any hidden neuron unreliable. The process is in contrast to standard backpropagation technique that builds up brittle coadaptations on the training set, reducing the capability to generalize to unseen data (testing set) [36]. The last layer has 18 units corresponding to the 18 chipless tag classes we use in this study. It uses SoftMax as the activation function to output probabilities of multiple categories, Y.

Our ML application was implemented in Python programming language, using the open-source neural network library Keras [37], a powerful collection of APIs that covers every step of the ML workflow. The subsequent preprocessing steps were performed in the same environment.

#### 2.3.1. Data Preprocessing

**Figure 10:**
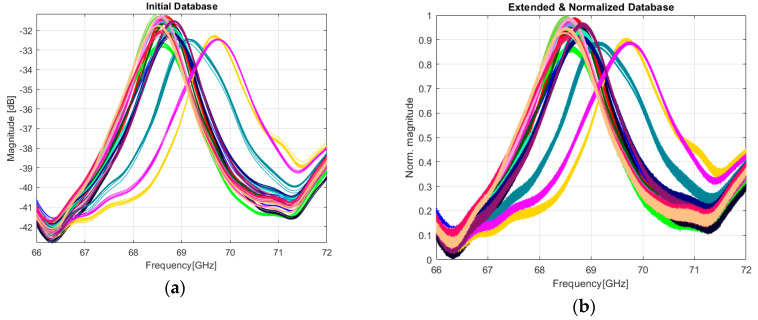
The result of extending and normalizing EM signatures. Each color is representative for a tag: (**a**) initial 180 EM signatures measured for 18 different tags; (**b**) The extended database with noised and normalized EM signatures.

Each tag was measured 10 times in the manner discussed in Section 2.2, meaning that we have a relatively small dataset to train a classifier. The initial database consisting of 10 × 18 = 180 measurements is presented in Figure 10a. In this case, the results might be unreliable and therefore an extended database is desired. Using the concept of data augmentation [38], we can generate more measurements derived from the original data by adding noise to an application-related extent. The objective is to improve accuracy and the robustness of the classifier. In our case, a natural way to accomplish data augmentation is to compute the IFFT of the EM signatures in Figure 10a, followed by adding AWGN while maintaining an SNR of 45–54 dB. Finally, we compute back the FFT of the noised time-domain signals. The resulting database contains now 200 measurements for each one of the 18 tags (200 × 18 = 3600 measurements). The addition of the white gaussian noise models a real scenario where the tag might not be in the direct visibility of the reader and, as a consequence, the EM spectrum of the back-scattered signal might differ in terms of amplitude. The most common situation in supply chains environment is when the product is packaged and is in different positions relative to the reader. Therefore, the augmentation technique increases the diversity of information stored in each EM signature, making each one of them reflect a possible situation in a real environment.

Another important step before the training process is to feed normalized data to a network. In general, normalizing the data speeds up the learning process and leads to faster convergence. The evaluation of the ML approach is conducted on the database presented in Figure 10b. The range of our data is mapped from (−42.8, −31.1) to (0, 1) using the following formula:

(1) $$X_{\mathrm{norm}}=\frac{X\;-\;\min\left(X\right)}{\max\left(X\right)\;-\;\min\left(X\right)}$$

#### 2.3.2. Neural Network Optimization

In order to find the optimal number of hidden units for our model we used the random search (RS) optimization approach. RS is empirically and theoretically demonstrated to be more efficient for hyper-parameter optimization than grid search or manual search [39]. A search space refers to the domain of the function that needs to be optimized (cost function). In our case, the search space is a priori defined as $M^{2}$, where M={32,40,…,128} units.

The RS algorithm randomly chooses a candidate solution to minimize the cost function. Until a termination criterion is met, a new candidate is sampled from the search-space, given a radius surrounding the initial solution. If the evaluation of the new candidate solution leads to a lower value, the algorithm moves to that position and starts over the process. Having a relatively small search-space, RS quickly found that 96 hidden units per layer are sufficient to learn from our data. The neural network tested further in the study has 96 units per hidden layer.

## 3. Results and Analysis

In the following section we firstly present the results of our best model that uses Adam [40] as stochastic optimization technique. The model’s capacity is demonstrated by monitoring its learning curves (LCs) and using the confusion matrix to enhance the visualization of the performance of the algorithm. A k-fold cross validation [41] is conducted to further confirm our results. In the end of this section, we provide a performance comparison firstly between optimization methods and secondly between different techniques found in the literature.

### 3.1. Neural Model Evaluation

The LCs provide a representation of the learning process over time. It is common to visualize the dual LCs using the training and validation dataset. The training LC emphasizes how well the model is learning, while the validation curve shows how well the model is generalizing. A model is capable of generalization if it successfully classifies other data than the data from the training set. Both metrics imply how the model behaves after each iteration. The loss is calculated with respect to the SoftMax function. The accuracy is determined by comparing the predictions of the neural model to the true data. The interpretation of the LCs can determine if the model is underfitting or overfitting. Overfitting means that a model has learnt the training dataset too well. This has implications in its generalization power. The more specialized a model becomes, the less capable it is to generalize to new data. Overfitting can be detected by analyzing the gap between the training and validation LCs. If the training loss is much lower than the validation loss over time, the model learnt the training observations by heart and cannot respond accordingly to other unseen observations. On the other hand, underfitting refers to an inadequate learning of the dataset. This can imply that the model does not have the necessary capacity to learn the complexity of the dataset, thus further tuning is required. Underfitting can be observed if the training LC shows a flat line or relatively high loss/low accuracy.

**Figure 11:**
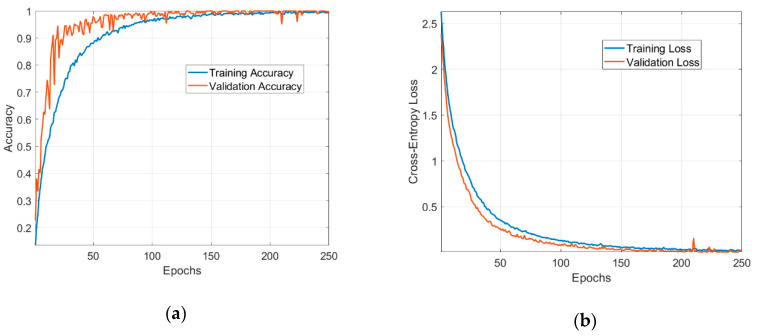
Metrics used in the evaluation of the neural network: (**a**) training and validation accuracy; (**b**) training and validation loss.

Figure 11 shows the evolution of the training and validation metrics (loss and accuracy) when using Adam as the update method for the network’s weights. To visualize the LCs, we fit our model on a training set that contains 2880 data samples and used the remaining 720 samples as the validation set. Other hyperparameters and options are mentioned as follows: the batch size used in the training is 32; the number of epochs was determined by monitoring the variance of validation loss over time; the average number of epochs for training is 250.

The model successfully learnt the dataset. The LCs have low fluctuations and a steady convergence, demonstrating an optimal fit. The validation steps performed at every epoch shows that the network is capable of generalization to unseen data. The validation accuracy goes up to 100% and demonstrates the ability of the ML approach to recognize the EM signatures of 18 different chipless tags.

**Figure 12:**
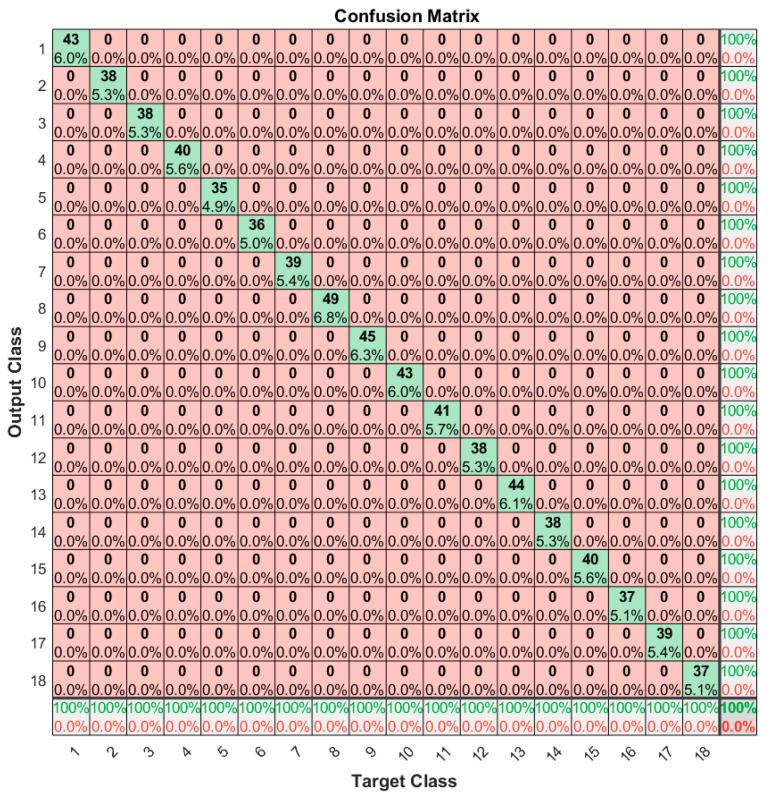
Confusion matrix for a separate test dataset.

To further enhance the visualization of the model performance, a confusion plot is presented in Figure 12. The confusion matrix makes it easy to see if the trained network is confusing classes between them (i.e., label the EM signature of one tag as the EM signature of another one). The rows correspond to the predicted tag class and the columns correspond to the target tag class. On the first diagonal, the numbers represent the measurements that are correctly classified. The off-diagonal cells are the misclassified EM signatures. The far-right column corresponds to the precision and false discovery rate (FDR), while the bottom row shows the recall and false negative rate (FNR). The cell in the bottom right shows the overall accuracy. Having the off-diagonal cells 0, the confusion plot implies that the classifier correctly predicted each EM signature, achieving 100% overall accuracy. The confusion plot can point out problems with the neural model by showing nonzero values outside the first diagonal. These problems might concern the datasets involved in the training or testing phase, insufficient number of epochs in training, small generalization capacity of the neural network, etc.

**Figure A1:**
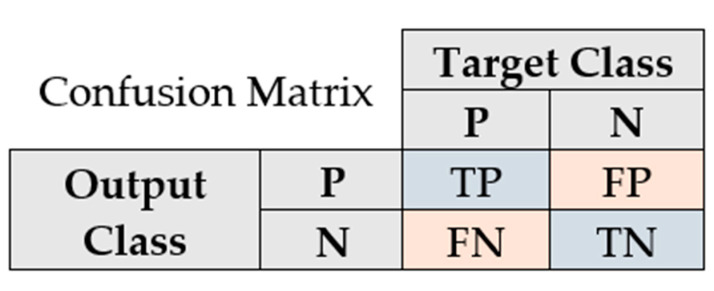
Confusion matrix described in abstract terms.

The diagonal cells show the number and the percentage of correct classifications by the network with respect to the dataset used for creating the matrix. For example, the first diagonal cell states that 43 EM signatures are correctly classified as being part of the same tag class. This number corresponds to 6.0% of all 720 EM signatures used for evaluation. The sum of numbers along each row represents the total number of measurements for the corresponding tag class. In order to interpret the metrics mentioned in the previous paragraph, we need to define some auxiliary terms. A true positive (TP) is a correct prediction of the positive class (P). A false positive (FP) is an incorrect prediction of the positive class. Similarly, a false negative (FN) is an incorrect prediction of the negative class (N). The positive class refers to the ground truth tag class, while the negative class refers to the other tag classes. Figure A1 in Appendix A shows the confusion matrix described in abstract terms. In this manner, we can now define the metrics used in the confusion plot and how they relate to each other:

(2) $$\;\mathrm{Precision}\;=\;\frac{\mathrm{TP}}{\mathrm{TP}\;+\;\mathrm{FP}}\;=\;1\;-\;\mathrm{FDR}$$

(3) $$\mathrm{FDR}\;=\;\frac{\mathrm{FP}}{\mathrm{TP}\;+\;\mathrm{FP}}=\;1\;-\;\mathrm{Precision}$$

(4) $$\mathrm{Recall}\;=\;\frac{\mathrm{TP}}{\mathrm{TP}\;+\;\mathrm{FN}}\;=\;1\;-\;\mathrm{FNR}$$

(5) $$\mathrm{FNR}\;=\;\frac{\mathrm{FN}}{\mathrm{TP}\;+\;\mathrm{FN}}\;=\;1\;-\;\mathrm{Recall}$$

As an example, we consider the outcomes of the measurements from the first tag class. The TP value is 43, FP is 0 (in the first row all cells except the first are 0), and FN is 0 (in the first column all cells except the first are 0). As a result, using the above formulas, the precision is 100%, the FDR is 0%, the recall is 100%, and the FNR is 0%.

### 3.2. K-Fold Cross Validation

Whenever we are designing ML algorithms, it is always a good practice to randomly split your data into training and testing sets. Still, this approach can miss valuable information about some examples because they were not used in training. To avoid losing important characteristics, it is useful to use k-folds cross validation [41]. The configuration parameter, k, defines the number of folds in which to split the dataset and its choice is mainly determined by the size of the dataset. The value of k is chosen such that each resulting fold is large enough to be statistically representative of the broader dataset. Another important characteristic in our application is to obtain an accurate estimator of the model’s performance. This implies that we have enough data for training and evaluating the model. Larger k reduces the size of the validation set and this leads to a less confident estimate of the model’s performance. In our case, 3 folds with 1200 measurements each represents a good choice taking into account our limited dataset and the number of classes it contains. We randomly split the data into 3 folds and cross-validate the neural network performance. In each one of the 3 cases, loss and accuracy metrics were monitored to evaluate the potential of the classifier. Table 2 shows the results. The cross-validation confirms the results in Section 3.1 by achieving a high recognition rate in all 3 cases. One thing worth mentioning is that the training dataset was reduced to 2400 measurements in this 3-fold evaluation. The model from Section 3.1 used a larger training set.

### 3.3. Performance Comparison

The following subsection presents a performance comparison for our model when it uses different optimization algorithms. The final paragraph provides an overview of the achievements of the most common techniques used to authenticate chipless RFID tags.

**Figure 13:**
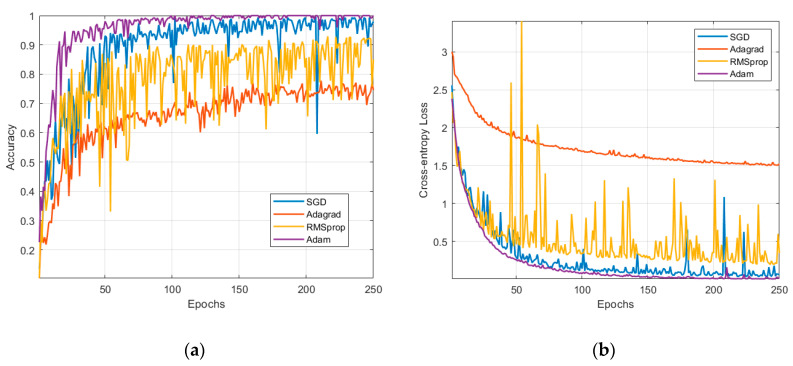
Comparison between Stochastic Gradient Descent (SGD), Adagrad, RMSprop, and Adam: (**a**) validation accuracy and (**b**) validation loss.

In Figure 13, different model update techniques were tested to compare the performances in terms of validation accuracy and loss. Appendix B briefly reviews these techniques. The Adam method outruns every other method and it is the preferred approach in this study. The second was Stochastic Gradient Descent (SGD), but in its case, the validation curves denote some unreliability coming from the dips and peaks in the metrics. To enhance SGD results, further regularization and optimization is needed. The third candidate is RMSprop with a lower recognition rate. The other techniques did not converge in the specified number of epochs; therefore, they are not suitable for our classification problem.

In Table 3, we summarized the recognition rate of different classification approaches found in literature, using different features of an RFID tag. The proposed method uses the EM signature of the V band chipless tags as the feature to train our model. The approach gave improved results over all the other cases. The low computational complexity of the neural network, along with having the fastest response to an input make our approach a better choice for the stated classification problem. Furthermore, as stated in Section 1, this approach has the opportunity to be synthesized and implemented in an IC. This represents a step forward towards developing a new generation of RFID fingerprinting systems that can be used to combat the counterfeiting problem of the manufactures.

## 4. Conclusions

This paper presents the actual context of the chipless tags authentication methods and proposes a ML approach in the characterization of the V band operating tags that can be used to solve the counterfeiting phenomena encountered by many industries.

Chipless RFID tags can generate a unique and unclonable identifier from its intrinsically random manufacturing process, thus making them suitable in authentication applications. The choice of using the V-band is argued by the fact that it better reflects the differences between EM signatures due to the smaller wavelength, which is comparable to the structural inhomogeneities of the designed tag. The inherent randomness of the fabrication process is visible in the EM spectrum in terms of phase and amplitude, therefore making them suitable to characterize the identity of each tag. This unique feature allowed us to successfully exploit the recognition power of ML.

The neural network used in the study has two hidden layers, uses ReLU activations, dropout regularization, and SoftMax loss. This architecture is chosen due to its simplicity and fast implementation. Moreover, it demonstrated that it is capable enough to train with the given data and to authenticate all tags with maximum accuracy. Another important aspect is related to the development of new fingerprinting equipment, that can be supported by integrating the neural network in an IC.

The proposed method is a low-cost and a high-level authentication approach in contrast to other available high secure authentication solutions (i.e., biological authentication or X-ray based solutions), where the cost of the equipment to examine the authenticity is high. The chipless tag can be inserted (or hidden) into the product and thus, it can be considered as a highly-secure seal.

Further study might include a generative adversarial network to extend our datasets, as opposed to the data augmentation technique. Moreover, instead of training with the EM signatures, modern instruments of nonlinear data analysis such as recurrence plot analysis (RPA) and recurrence quantification analysis (RQA) can lead to other unique characterizations of chipless RFID tags.

## Figures and Tables

**Table 1:** Similarities between simulated models on different operation frequencies.

Frequency	$E_{1}$ $;\;(E_{1}-17.5\;\mu m)$	$E_{1}-17.5\;\mu m;$ $E_{1}-35\;\mu m$	$E_{1};\;E_{1}-35\;\mu m$
X-band	0.9966	0.9971	0.9875
V-band	0.6059	0.6031	0.5750

**Table 2:** Three-fold cross validation loss and accuracy metrics.

Model ^1^	Loss	Accuracy (%)
Model 1	0.0208994	100%
Model 2	0.0371103	100%
Model 3	0.0369607	100%

^1^ A model trains on two folds and it is tested on the third fold. A fold has 1200 measurements.

**Table 3:** Recognition rate for different supervised and unsupervised methods.

Classification Technique	Recognition Rate
Euclidean Distance	92.12%
Normalized Correlation	91.97%
Lorentzian Distance	91.33%
Manhattan Distance	96.06%
ML with LDA	98.44%
Dynamic Time Warping	100%
Wavelet Transform Manhattan Distance	100%
Our approach	100%

## Appendix B

We formally present all the gradient update techniques used in our study.

- SGD is a simple and robust algorithm where the gradient of the cost function with respect to the weights, $\nabla_{\theta_{t-1}}f\left(\theta_{t-1}\right)$, is computed, and a fraction, η, of that gradient is subtracted off of the weights:
  **Algorithm A1: SGD Algorithm**
  $\begin{array}{l} g_{t}\leftarrow \nabla_{\theta_{t-1}}f\left(\theta_{t-1}\right)\\ \theta_{t}\leftarrow \theta_{t-1}-\eta g_{t} \end{array}$
- Adaptive Subgradient Descent (AdaGrad) divides η of every step by the $L_{2}$ norm of all previous gradients. The method stabilizes the model’s representation of common features and allows it to learn the rare ones.
  **Algorithm A2: Adagrad Algorithm**
  $\begin{array}{l} g_{t}\leftarrow \nabla_{\theta_{t-1}}f\left(\theta_{t-1}\right)\\ n_{t}\leftarrow n_{t-1}+g_{t}{}^{2}\\ \theta_{t}\leftarrow \theta_{t-1}-\eta \frac{g_{t}}{\sqrt{n_{t}+\varepsilon }} \end{array}$
- RMSprop replaces the sum in $n_{t}$ with a decaying mean parameterized by v. It solves the halting training of AdaGrad.
  **Algorithm A3: RMSprop Algorithm**
  $\begin{array}{l} g_{t}\leftarrow \nabla_{\theta_{t-1}}f\left(\theta_{t-1}\right)\\ n_{t}\leftarrow vn_{t-1}+\left(1-v\right)g_{t}{}^{2}\\ \theta_{t}\leftarrow \theta_{t-1}-\eta \frac{g_{t}}{\sqrt{n_{t}+\varepsilon }} \end{array}$
- Adam method combines classical momentum with RMSprop to improve the advantages of both algorithms. Bias correction terms m and n are initialized to 0.
  **Algorithm A4: Adam Algorithm**
  $\begin{array}{l} g_{t}\leftarrow \nabla_{\theta_{t-1}}f\left(\theta_{t-1}\right)\\ m_{t}\leftarrow \mu m_{t-1}+\left(1-\mu \right)g_{t}\\ \hat{m_{t}}\leftarrow \frac{m_{t}}{1-\mu^{t}}\\ n_{t}\leftarrow vm_{t-1}+\left(1-v\right)g_{t}{}^{2}\\ \hat{n_{t}}\leftarrow \frac{n_{t}}{1-v^{t}}\\ \theta_{t}\leftarrow \theta_{t-1}-\eta \frac{\hat{m_{t}}}{\sqrt{\hat{n_{t}}+\varepsilon }} \end{array}$
